# Survey based investigation into general practitioner referral patterns for spinal manipulative therapy

**DOI:** 10.1186/2045-709X-21-16

**Published:** 2013-05-29

**Authors:** Annabel Kier, Matthew George, Peter W McCarthy

**Affiliations:** 1 Welsh Institute of Chiropractic, University of South Wales, Treforest, Pontypridd CF38 2TE, UK; 2 Private Practice, Worthing BN11 1PS, UK

**Keywords:** Spinal manipulative therapy, Referral and personal usage by general practitioners

## Abstract

**Background:**

In the UK Physiotherapy, Chiropractic and Osteopathy are all statutory regulated professions. Though guidelines have supported the use of Spinal Manipulative Therapy (SMT) for low back pain (LBP), General Practitioners (GP) referral patterns to the 3 registered professions that perform SMT are generally unknown.

**Method:**

A short questionnaire was designed and piloted. Demographic information, patient referral to SMT and the GPs own personal utilisation of SMT were obtained. 385 GP’s were contacted representing approximately 20% of the GP’s in Wales Autumn 2007.

**Results and discussion:**

182 (50.8%) completed questionnaires were returned.

*Profile characteristics*: 2/3 of respondents were male, 79% were 40 years old or older (statistically reflective of the total population of GPs in Wales at that time) and 62% had 20 years or less in practise. *Personal use of SMT by GP’s*: 48 respondents had sought SMT treatment and a further 56% of those that had not previously sought SMT indicated that they would consider doing so. *Patient referral to SMT by GP’s:* 131 respondents (72%) had referred patients to SMT and of those who had not a further 13% would consider referring. The general referral pattern and utilisation pattern was Physiotherapy: Osteopathy: Chiropractic. 21% who had never referred patients neither had, nor would consider it for themselves. A small subgroup appeared to manage personal choice differently from patient referral: 5 individuals who had not referred patients either had or would consider it for themselves and 23 of the group that would refer patients neither had nor would seek it for themselves.

**Conclusions:**

This limited investigation indicates that GP’s do practise consistently with guidelines on back pain and utilise SMT as a care option. Although the main option for referral was physiotherapy, slightly over 40% of respondents who expressed a preference would refer to either osteopathy or chiropractic, or both in preference to physiotherapy. There was a small proportion that did not and would not refer patients for SMT regardless of personal use of SMT; these suggested use of acupuncture. Further investigation is needed to determine the alternatives to SMT offered to patients and the decision-making criteria for patient referral to subtypes of SMT practitioner.

## Background

There has been a widened use of Complementary and Alternative Medicine (CAM) over the last two decades [1-7]. One area where this change is most noticeable is that of Low Back Pain (LBP).

LBP is a commonly experienced symptom, which approximately 38% of adults will have in any one year [8]. Of these, 1 in 4 LBP sufferers experience significantly disabling symptoms resulting in a considerable cost to society in particular and a significant proportion of the caseload is dealt with in primary care thus challenging both the health care system and the General Practitioner (GP). Recent research outcomes and guidelines have supported the use of Spinal Manipulative Therapy (SMT) for this condition [9-15]. Although SMT is available in the UK from a small number of GPs the main provision is accessed via 3 statutory registered professions, Chiropractors, Osteopaths and certain qualified Physiotherapists [16]: statutorily regulated by the General Chiropractic Council (GCC), the General Osteopathic Council (GOC) and The Health Professions Council (HPC- Physiotherapists), respectively. Although these three professions have traditionally appeared to have little common ground, anecdotally it appears that some degree of synergy exists between them regarding the management of back pain, as referenced in the guidelines: even though there may be differences in the choice and utilisation of certain manipulative procedures, rehabilitation and exercise protocols [16]. The engagement of services from these private practitioners by the National Health Service (NHS) is still an ardently debated topic [17]. This work has the potential to act as a baseline study for future investigations endeavouring to determine the impact of change on engagement of these services for Low Back pain.

Although the situation regarding direction of GPs to services by guidelines specifically for LBP has been present for a number of years [11], the proportion of GPs who either do, or would consider referring patients to SMT practitioners, in compliance with the low back pain guidelines, is unknown. Similarly there is little information regarding GP’s referral preference towards a specific type of SMT or the GP’s personal utilisation of SMT and whether these two aspects are linked.

## Method

A short questionnaire was designed and initially piloted internally for face/construct validity within the university and a sample of GPs (personal connection). The questionnaire requested information regarding a GP’s age, sex, years spent practising, patient referral to SMT as well as the GPs own personal utilisation of SMT. Where there may not have been a need/wish to seek SMT, the GP was asked to answer the question hypothetically. No other personal information was requested. A covering letter containing information and invitation to complete the questionnaire as well as a stamped and addressed return envelope was sent to each selected individual. Anonymity of participants was maintained by the destruction of all returned envelopes once they had been opened and separated from the questionnaire, thereby eliminating the potential of identifying participants from the postmark.

The Primary Care Trust (PCT), PCT sub-division and GP name and address information was obtained from http://www.specialistinfo.com (2007) [18], an internet directory of over 36,800 hospital consultants and over 38,000 GPs in the UK. Any qualified GP practising in Wales at the time of the study and whose name was listed on the website was eligible for inclusion the study. A selection of 358 GPs from all 22 Primary Care Trusts in Wales were approached for the study representing approximately 20% of the then 1749 practising GP’s in Wales during Autumn 2007 who were available on the chosen database. Potential participants were selected by sampling each GP name from alternating sub-divisions of the PCTs until the required number were selected. The selection process was otherwise random, with no consideration made for other factors such as male:female ratio. The study had received approval by the Welsh Institute of Chiropractic research unit ethical review group of the Chiropractic division, University of Glamorgan. In order to reduce bias in terms of SMT selection, no indication of the involvement of a chiropractic teaching facility remained on any of the literature. The return address was to the Faculty of Health, Sports and Science.

The data is presented as either raw numerical or frequency distributions. Limited statistical analysis was performed using Chi^2^ test to compare the distributions across groups.

## Results

Of the original 358 posted questionnaires, 187 were returned, however 5 were disregarded as void due to completion errors. The remaining 182 (50.8%) were analysed and are reported below.

### Profile characteristics

Two thirds 118 (64.8%) of the 182 respondents, were male compared to 64 (35.2%) female. Seventy nine percent of respondents (143/182) were forty years of age or older (105 male and 39 female) in contrast to twenty one percent younger than 40 years of age (13 males and 25 female). Regarding years of experience in practice, sixty two percent had twenty or less years experience (71 male and 42 female) and thirty eight percent had more than twenty years experience (47 male and 22 female).

### Personal use of SMT by GP’s

48 respondents’ forming 26.4% of the 182 GPs who returned the completed questionnaire had personally sought treatment from a SMT practitioner: of which 32 were male in the 30–59 age range, mainly in the 40–49 and 50–59 age groups, and 16 were female between 20–59 years of age, mainly in the 30–39, 40–49 and 50–59 age groups (there was no difference between these populations: Chi^2^ p>0.1).

The remaining 134 GPs of the 182 returned for analysis, had not previously sought SMT. Of this group, 75 (56%: comprising of 45 males and 30 females) responded that they would consider seeking treatment from a SMT practitioner in the future. The males were mostly in the 40–59 year age range; whereas the females were mostly in the 30–39 age range suggesting age was not an influencing factor in this group. In total, 123 (68%) GPs indicated that they either had personally sought or would consider seeking SMT, leaving 59 (32%) that neither had, nor would consider, seeking SMT treatment in the future, this group appeared no different from the main population in relation to gender or age range (Chi^2^ p>0.1).

### Patient referral for SMT by GP’s

131 respondents (72%), 88 males and 43 females, indicated that they had referred a patient to a SMT practitioner. Of these, 31 of the males and 14 of the females had themselves utilised SMT with an additional 40 males and 23 females indicating they would personally consider utilizing SMT in the future. However of this subgroup of GP’s, in apparent contrast to their treatment of patients, 17 of the 88 males and 6 of the 43 females had not personally used, and responded that they would not consider utilising SMT themselves in the future. Unfortunately, the structure of the questionnaire did not allow any analysis of the number of GPs who had referred patients but would not do so in the future. For those categories where a data was available (age and gender), none of these distributions varied significantly from the population statistics of those GPs who responded to the questionnaire (Chi^2^ p>0.1).

Of the 51 (29%) remaining GPs (30 male and 21 female) who had not previously referred a patient to a SMT practitioner, 13 (7%), comprising 6 males and 7 females, indicated they would consider referring a patient to a SMT practitioner in the future (Chi^2^ p>0.1). This left 38 (21%) of the respondents (24 males and 14 females) who indicated that they have not and would not consider referral of their patients to a SMT practitioner.

Therefore, of the 182 respondents, 144 (79%) indicated that they already had or would consider referring a patient to a SMT practitioner. For outline of all subdivisions see Table 1.

**Table 1:** Preference regarding SMT (referral and/or personal usage)

**Respondents**	**Number of completed questionnaires**	**Have used SMT previously**	**Have not used**	**Have not used but would consider using SMT**	**Total having either used SMT or would consider its use personally**	**Those GPs who have either used SMT ( *or would consider using SMT *) personally**			**GPs that have not used and would not seek SMT personally (non-users)**	**GPs that have not used SMT personally ( *and GPs who would not consider use of SMT *)**		**Total of those who either have ( *or would consider *) referring patients to SMT**
						**Have referred patients to SMT**	**Have not referred patients to SMT but would consider referring patients in the future**	**Have not referred nor would consider referring to SMT**		**Have referred or would consider referring patients to SMT**	**Have not referred and would not consider referring to SMT**	
**Male**	118	32	86	45	77	31+(*40*)=71	1+(*4*)= 5	0+(*1*)= 1	41	17+(*1*)=18	23	105+(*7*)= 112
**Female**	64	16	48	30	46	14+(*23*)=37	0+(*5*)= 5	2+(*2*)= 4	18	6+(*2*)= 8	10	48+(*10*)= 58
**Total**	182	48	134	75	123	45+(*63*)=108	1+(*9*)= 10	2+(*3*)= 5	59	23+(*3*)=26	33	153+(*17*)=170

Analysis of results from respondents to the questionnaire and the subdivisions relating to gender, personal utilisation off and patient referral to SMT. In the sections describing the groups that either have or have not previously used SMT, the data presented outside the parentheses refers to those who have experienced and within parentheses relates to those who have not yet experienced SMT.

### Choice of SMT practitioner in context of personal usage by GPs

When asked to name the type of therapist that GPs had personally sought or would consider seeking SMT treatment from, the results were as follows: physiotherapist 22% / osteopath 16% / chiropractor 13%. Interestingly, those who had previously sought SMT treatment appeared to distribute significantly differently (P<0.01) to those who had expressed a preference but had not already experienced SMT treatment (Table 2). Where preference was indicated, the order was found to be similar for both males and females. A further 23% responded that they had no preference and would choose to seek help from either of the 3 professions listed. 24% of respondents were selective in a different manner in that they elected to choose 2 of the 3 options; with osteopathy being favoured in combination with either chiropractic or physiotherapy. A small subgroup (n=4) indicated that they would choose SMT from a profession other than those proposed in this study (this population was not included in Table 2).

**Table 2:** GP’s previous and potential future personal usage of SMT

	**Expressed preference for personal use**		**Total expressing personal preference**	**Expressed preference for Patient referral/recommendation**		**Total expressing preference for referral**
	**Previously**	**Future**		**Previously**	**Future**	
Osteopathy	10(21%)	10(13%)	***20(16%)***	15(11%)	1(8%)	***16(11%)***
Chiropractic	13(27%)	3(4%)	***16(13%)***	9(7%)	1(8%)	***10(7%)***
Physiotherapy	9(19%)	18(24%)	***27(22%)***	18(14%)	8(62%)	***26(18%)***
No preference	6(13%)	22(29%)	***28(23%)***	35(27%)	1(8%)	***36(25%)***
Osteopathy /Chiropractic	6(13%)	7(9%)	***13(11%)***	28(21%)	1(8%)	***29(20%)***
Chiropractic/ Physiotherapy	0	4(5%)	***4(3%)***	9(7%)	0	***9(6%)***
Osteopathy/Physiotherapy	3(6%)	9(12%)	***12(10%)***	13(10%)	1(8%)	***14(10%)***
Other	1(2%)	2(3%)	***3(2%)***	4(3%)	0	***4(3%)***
*n=*	*48*	*75*	***123***	*131*	*13*	***144***

Preference for services: either used previously or not used previously with the caveat that they may do so in the future- regarding SMT and patient referral or recommendation. Percentages are presented for comparison, please note the numbers have been rounded to integers and so may not summate to 100%.

### Choice of SMT practitioner by GP’s in context of patient referral

A similar ratio to that above was found for GP’s referral preference for their patients: physiotherapist 19% / osteopath 11% / chiropractor 7%. Interestingly 42% (of respondents who had reported a preference: n=127) did so by choosing either osteopath, chiropractor or both from the 3 options offered. A further subgroup of responses (30%) was most reflective of the NICE guidelines by indicating they have or would refer/recommend their patients to (any) either of the 3 professions. In conclusion, some GP respondents were very specific in their choice of which SMT practitioner they would refer their patients to. As with personal use, some opted for 2 of the 3 professions. In this case the preferred combinations favoured osteopathy (with either chiropractic or physiotherapy: see Table 2). A small subgroup of 3: (not included in Table 2) gave unsolicited information in that they would refer to someone other than SMT practitioners and all indicated Acupuncture as their choice. Though personal comments were not actively sought on the questionnaire one respondent indicated that choice of referral would depend on the practitioner and not the profession, thereby highlighting that personal knowledge of the practitioner was considered at least as important as type of SMT practitioner. Another practitioner emphasised that though they neither utilise for themselves nor refer patients, the option of self-referral is made clear to their patients.

## Discussion

On the whole the results of this small study suggest that 140 (77%) of the 182 GP respondents had either already referred patients to SMT practitioners or would consider doing so in the future. Should this be a true representation of the picture across GPs in Wales, the results indicate that over 3 quarters of the GP population were practising consistently with the current guidelines [14]. However, this study’s focus on SMT might have under-represented the whole story as a proportion of GPs may refer to other modalities covered by the guidelines, which were not specifically asked for in this study. Although Physiotherapy was the SMT provider of choice for most GPs who expressed a preference, there was a reasonably large proportion (42%) that indicated preference for either osteopathy or chiropractic, or both.

The sample size of this present study is admittedly small, which may have affected some of the statistical analysis of the small sub-groups. Although the total GP population in the region at the time was 1749, the sample reported here was obtained consistently with sampling procedures used in similar studies such as Corbett et al. in 2009 [19]. The literature varies regarding what constitutes a “good” or appropriate survey response rate. Variables such as population size, how the response is collected (interview, phone calls or mail-out) and whether a survey relates to individuals or organizations, appear to affect the response, as well as response rate [20]. In this case, the overall response rate was 50.8%, which although could be considered good [19] some would think it adequate [20], and others unacceptable with one issue being non-response error [21,22]. Although, as a population, GP response rate to questionnaires has been interpreted as being generally low by McAvoy & Kaner [23], due to factors such as lack of information, perceived relevance and or feedback, our study appears to have a high response rate. This might suggest that the topic is one of interest to GPs within this catchment area. In addition to topic, attempts were also made to circumvent elements of potential bias by not mentioning any professional affiliation in the questionnaire, the accompanying literature or mail address.

Regarding response bias, although the data presented here appears to show differences in distribution regarding gender and age, these do generally appear reflective of the total population at the time [24]. For instance, there were nearly twice the number of male respondents to this present survey than female; as at the time the population of GPs in Wales was approximately 60% male [24], therefore, this response would not appear to be too biased in relation to gender. Most male respondents were between 40–59 year of age and most females were between 30–39 years old, which is also in line with the general population statistics for Welsh GPs in 2007–9 [24]. Regarding the whole sample of respondents, 79% were 40 years of age or older, comprising 3 times as many males as females. The statistics available do not allow completely accurate comparison (the data presented as 30–44 and 45-above: [24]), however in the population, 62% were above 45 yrs of age, of which there were approximately 2.4 times as many males to females. In comparison, those less than 40 years of age were represented by double the amount of females compared to males: again the population statistics show 1.3 times the number of females to males [24].

Of the whole sample, 62% had less than 20 years experience, and numerically, was approximately equally distributed between the sexes. In contrast, approximately twice the number of males to females had had greater than 20 years experience. Of the 48 respondents that had utilised SMT, one third were female, which was proportionate with the male:female distribution in the overall response. As there is no information available regarding which point in their lives the participants sought their care, it is impossible to elaborate upon this in relation to trends regarding health care sought in the general population. In hindsight this may have been interesting to pursue and should potentially be considered for any future surveys of this kind.

A higher percentage of males (74% of the males) had referred patients to SMT compared to females (65%) in apparent contrast to previous studies that showed that female general practitioners tend to refer to CAM more than their male counterparts [4,25,26]. Although there is no data available, this finding might simply relate to the disproportionate distribution of males and females in the older age range of GPs. Of the small proportion of individuals (n=38/182: 21%) who would not consider referring patients for SMT, 33 neither have, nor would consider utilising SMT for themselves. Although this appears inconsistent with the guidelines, it does potentially indicate a level of personal choice being reflected in the decision making process concerning patient referral to SMT. Although there are no other studies of this exact nature for direct comparison, research performed prior to guideline direction on SMT reported a correlation between personal use and referral to CAM therapies by medical practitioners [4,27-32]. Furthermore, Brien et al. [31] reported that most GP’s would consider referral via NHS contract to CAM therapies where there are no other therapeutic options for their patients. Various factors, including clinical evidence, appear to increase the likelihood of referral, but in particular the individual GP’s positive attitude to and experience of CAM, including a trusting relationship with the CAM practitioner and the patients attitude toward CAM were reported as major factors in the decision making process. In the Brien et al. [31] study, 71.4% of the respondents indicated that they had referred a patient to a CAM practitioner with a further 7% indicating that they would consider it in the future, results which compare favourably with those reported here.

Of the remaining 5 of those 38 respondents in this study who would not refer to SMT, it was not unexpected to find 2 had experienced it for themselves. However, it was interesting and unexpected to find that the remaining 3 of the 5 would consider SMT for themselves, although they would not refer their patients. Therefore it appears that there are a number of respondents in each category suggesting they would manage patients with LBP contrary to their own personal choice. Albeit a small proportion, this would indicate that personal choice does not always affect compliance with guidelines or the inclination to refer. The exact reasoning for these differences was not explored here.

Though the National Institute for Health and Care Excellence (NICE) guidelines are exactly that, advice given on what is considered best practice at the time; it is open to personal interpretation by a practitioner based on their assessment and perception of the individual needs and choice of a patient. Having said that, many of the GPs surveyed in this study, appear to be following the guidelines. 96% (118 of 123) of those that had sought or would consider seeking SMT for themselves would refer patients as would 44% (26 of 59) of those GPs who have not and would not consider seeking SMT for themselves. Unfortunately, however, it is not possible to assess whether the proportion referring to SMT may have been affected by the presence of guidelines.

A number of studies have investigated the compliance of various physician groups to National and International guidelines [33-37]. Though not exhaustive, Table 3 lists several factors that may influence the physician’s decision making process, besides any previous positive/negative communication with SMT practitioners [4,30,38-43].

**Table 3:** Factors that might influence a GP’s responses to a questionnaire regarding complementary and alternative medicine


Age	Family behaviour	Personal views
Availability - SMT providers	Finances of patient	Public/Private sector
Colleagues’ attitudes	Gender of provider	Space/facilities
Education	Liability/legal issues	Specialisation
Ethnicity	Management	(The) State
Evidence	Media	Status of provider
Experience - in years	Practice size	Strategic reasons
Familiarity - with SMT	Patient preference	Time

Factors that might have influenced the questionnaire responses from the GP respondents in this study. The information derives from a previous study that assessed CAM rather than SMT specifically. (Adapted from Hirschkorn & Bourgeault, [43]).

It has been suggested that the basis for lack of compliance to guidelines includes factors such as; concerns about loss of autonomy, guidelines being “too rigid”, oversimplification of the guidelines and their production being “motivated by a desire to cut costs” [36,37,44]. In addition, although a GP may have been in agreement with the guidelines, these are not necessarily congruent with patients’ wishes. Non-adherence to guidelines and contradictory information supplied to patients by other professionals should also be considered important barriers to guideline adherence [33,36,37].

The understanding of why and to which SMT or CAM practitioner GPs may refer is neither transparent nor necessarily consistent across the profession. It has been suggested that unnecessary jargon could and does negatively affect communication and, therefore, the GP – CAM- and possibly SMT- practitioner relationship [38,43,45]. It is worth noting that the study presented here also allowed GPs to indicate their tendency to refer to combinations of SMT practitioner. Although, the underlying reasoning for a GP’s selection of SMT practitioner cannot be deduced from this survey, personal conversations and anecdote points to the availability of physiotherapy on the NHS being an important factor; as osteopathy and chiropractic care are mostly found in the private sector. Therefore, patients may benefit from greater inter-profession dialogue and enhanced patient choice available from the NHS: complying with best practice regarding patient-centred care. Currently, the main issues which can impede this process are those of lack of quantity of qualified and registered practitioners, lack of understanding of low back pain subgroups and appropriate referral mechanisms to discussed treatment modalities [46]. A further limitation of the study would be the lack of information underlying the individual GP’s choices regarding personal utilisation of and patient referral for SMT. Should a follow up study be conducted inclusion of such considerations would be recommended.

## Conclusion

The results presented here indicate that a high proportion of the GP’s responding to this questionnaire did or would refer patients for SMT, which is in contrast to anecdotal belief amongst some SMT practitioners and previous research findings [47]. This study found that when referrals occur to a specific profession, the majority of patient referrals were to physiotherapists. This result mirrors a previously reported outcome from a study of GPs’ perception regarding which of the three types of SMT were considered to be most useful: physiotherapy>osteopathy >chiropractic [48].

Although limited, this survey has allowed for an insight into the GP’s utilisation and referral patterns to practitioners of SMT in Wales. Further investigation is needed to determine the alternatives to SMT offered to patients and the decision-making criteria for patient referral to subtypes of SMT practitioner.

## Abbreviations

CAM: Complementary and alternative medicine; LBP: Low back pain; GP: General practitioner; SMT: Spinal manipulative therapy; GCC: General chiropractic council; GOC: General osteopathic council; HPC: Health professions council; NHS: National health service; PCT: Primary health care trust; NICE: National institute for health and clinical excellence.

## Competing interests

The authors declared that they have no competing interests.

## Authors’ contributions

MG compiled initial idea of area of interest and partook in the development of the questionnaire. Also participated in the initial write up of the data collected. AK assisted in development of the idea and design of questionnaire. Supervised the initial write up of this data, authored the final paper and in-depth analysis of data collected. PWM assisted in development of the initial questionnaire design, co-authored the final paper and in-depth analysis of data collected. All authors read and approved the final manuscript.

## References

[B1] White A Ernst E Economic analysis of complementary medicine: a systematic review Complement Ther Med 2000 8 111 118 10.1054/ctim.2000.0356 10859604

[B2] Harris P Rees R The prevalence of complementary and alternative medicine use among the general population: a systematic review of the literature Complement Ther Med 2000 8 88 96 10.1054/ctim.2000.0353 10859601

[B3] Thomas KJ Nicholl JP Coleman P Use and expenditure on complementary medicine in England: a population based survey Complement Ther Med 2001 9 2 11 10.1054/ctim.2000.0407 11264963

[B4] Lewith GT Hyland M Gray SF Attitudes to and use of complementary medicine among physicians in the United Kingdom Complement Ther Med 2001 9 167 172 10.1054/ctim.2001.0475 11926430

[B5] Emslie MJ Campbell MK Walker KA Changes in public awareness of, attitudes to, and use of complementary therapy in North East Scotland: surveys in 1993 and 1999 Complement Ther Med 2002 10 148 153 10.1016/S0965229902000663 12568143

[B6] Schmidt K Jacobs PA Barton A Cross-cultural differences in GPs’ attitudes towards complementary and alternative medicine Complement Ther Med 2002 10 141 147 10.1016/S0965229902000560 12568142

[B7] Haetzman M Elliott AM Smith BH Hannaford P Chambers WA Chronic pain and the use of conventional and alternative therapy Fam Pract 2003 20 147 154 10.1093/fampra/20.2.147 12651788

[B8] Bishop A Foster N Thomas E Hay E How does the self-reported clinical management of patients with low back pain relate to the attitudes and beliefs of health care practitioners? A survey of UK general practitioners and physiotherapists Pain 2008 135 187 195 10.1016/j.pain.2007.11.010 18206309 PMC2258319

[B9] Meade TW Dyer S Browne W Townsend J Frank AO Low back pain of mechanical origin: randomised comparison of chiropractic and hospital outpatient treatment Br Med J 1990 300 6737 1431 1437 10.1136/bmj.300.6737.1431 2143092 PMC1663160

[B10] Meade TW Dyer S Browne W Frank AO Randomised comparison of chiropractic and hospital outpatient management for low back pain: results from extended follow up Br Med J 1995 311 7001 349 351 10.1136/bmj.311.7001.349 7640538 PMC2550426

[B11] Royal College of General Practitioners Clinical guidelines for the management of acute low back pain 1999 Available http://www.chiro.org/LINKS/GUIDELINES/FULL/Royal_College/index.html

[B12] NICE Clinical guidelines IPG 183 2006 http://www.nice.org.uk/nicemedia/live/11204/51670/51670.pdf

[B13] Department of Health Musculoskeletal Services Framework 2006 available at http://www.susanoliver.com/pdf/MSF_Final.pdf

[B14] NICE Clinical guidelines CG88 2009 available at http://www.nice.org.uk/nicemedia/live/11887/44343/44343.pdf

[B15] Koes BW van Tulder M Chung-Wei CL Macedo LG McAuley J Maher C An updated overview of clinical guidelines for the management of non-specific low back pain in primary care Eur Spine J 2010 19 2075 2094 10.1007/s00586-010-1502-y 20602122 PMC2997201

[B16] Harvey E Burton AK Moffett JK Breen A Spinal manipulation for low-back pain: a treatment package agreed by the UK chiropractic, osteopathy and physiotherapy professional associations Man Ther 2003 8 46 51 10.1054/math.2002.0472 12635637

[B17] Hartvigsen J Foster NE Croft PR We need to rethink front line care for back pain Br Med J 2011 342 d3260 10.1136/bmj.d3260 21613346

[B18] Filed under Wales, Practices within PCT’s 2008 http://www.specialistinfo.com (specialist info.com/a_directory_gp2.php)

[B19] Corbett M Foster NE Ong BN GP attitudes and self-reported behaviour in primary care consultations for low back pain Fam Pract 2009 26 359 364 10.1093/fampra/cmp042 19546118 PMC2743733

[B20] Babbie E Survey research methods 1990 Belmont, CA: Wadsworth

[B21] Schutt RK Investigating the social world; the process and practice of research 1999 2 Thousand Oaks, CA: Pine Forge Press

[B22] Hager MA Wilson S Pollak TH Rooney PM Response rates for mail surveys of nonprofit organizations: a review and empirical test Nonprofit and Voluntary Sector Quarterly 2003 32 252 267 10.1177/0899764003032002005

[B23] McAvoy BR Kaner EF General practice postal surveys: a questionnaire too far? Br Med J 1996 313 732 10.1136/bmj.313.7059.732 8819446 PMC2352115

[B24] Swain K General Medical Practitioners in Wales 2002 to 2012, First release SDR 47/2013 The Health and Social Care Information Centre Available from: http://wales.gov.uk/topics/statistics/headlines/health2013/general-medical-practitioners-2002-2012/?lang=en

[B25] Schachter L Weingarten MA Kahan EE Attitudes of family physicians to nonconventional therapies: a challenge to science as the basis of therapeutics Archive of Family Medicine 1993 2 1268 1270 10.1001/archfami.2.12.1268 8130909

[B26] Botting DA Cook R Complementary medicine: knowledge, use and attitudes of doctors Complement Ther Nurs Midwifery 2000 6 41 47 10.1054/ctnm.1999.0437 11033653

[B27] Reilly DT Young doctors’ views on alternative medicine Br Med J 1983 287 6388 337 339 10.1136/bmj.287.6388.337 6307463 PMC1548552

[B28] Borkan J Neher JO Anson O Smoker B Referrals for alternative therapies J Fam Pract 1994 39 545 550 7798857

[B29] Wharton R Lewith G Complementary medicine and the general practitioner Br Med J 1986 2920 6534 1497 1500 10.1136/bmj.292.6534.1498 PMC1340501 3087494

[B30] Jump J Yarbrough L Kilpatrick S Cable T Physicians’ attitudes toward complementary and alternative medicine Integrative Medicine 1998 1 149 153 10.1016/S1096-2190(98)00038-9

[B31] Brien S Howells E Leydon GM Lewith G Why GPs refer patients to complementary medicine via the NHS: a qualitative exploration Primary Health Care Research & Development 2008 9 205 21523716803

[B32] Sewitch MJ Cepoiu M Rigillo N Sproule D A literature review of health care professional attitudes toward complementary and alternative medicine Practice Review 2008 13 139 154

[B33] Hayward RSA Guyatt GH Moore K-A McKibbon KA Carter AO Canadian physicians’ attitudes about and preferences regarding clinical practice guidelines Can Med Assoc J 1997 156 1715 1723 9220923 PMC1227586

[B34] Bishop P Badii M Wing P Implementation of clinical practice guidelines in workers compensation board patients with acute mechanical back pain: a prospective randomized trial Spine J 2002 2 5, Supplement 62 63

[B35] Barry CA The role of evidence in alternative medicine: contrasting biomedical and anthropological approaches Soc Sci Med 2006 62 2646 2657 10.1016/j.socscimed.2005.11.025 16376470

[B36] Fullen BM Maher T Bury G Tynan A Daly LE Hurley DA Adherence of Irish general practitioners to European guidelines for acute low back pain: a prospective pilot study Eur J Pain 2007 11 614 623 10.1016/j.ejpain.2006.09.007 17126046

[B37] Chenot JF Scherer M Becker A Donner-Banzhoff N Baum E Leonhardt C Keller S Pfingsten M Hildebrandt J Basler H-D Kochen MM Acceptance and perceived barriers of implementing a guideline for managing low back in general practice Implementation Science 2008 3 7 12 10.1186/1748-5908-3-7 18257923 PMC2275295

[B38] Brussee WJ Assendelft WJJ Breen AC Communication between general practitioners and chiropractors J Manipulative Physiol Ther 2001 24 12 16 10.1067/mmt.2001.112016 11174690

[B39] Astin JA Marie A Pelletier KR Hansen E Haskell WL A review of the incorporation of complementary and alternative medicine by mainstream physicians Arch Intern Med 1998 158 2303 2310 10.1001/archinte.158.21.2303 9827781

[B40] Berman BM Bausell RB Hartnoll SM Beckner M Bareta J Compliance with requests for complementary-alternative medicine referrals: a survey of primary care physicians Integrative Medicine 1999 2 11 17 10.1016/S1096-2190(99)00014-1

[B41] Hasan MY Das M Behjat S Alternative medicine and the medical profession: views of medical students and general practitioners East Mediterr Health J 2000 6 25 33 11370337

[B42] Perkin MR Pearcy RM Fraser JS A comparison of the attitudes shown by general practitioners, hospital doctors and medical students towards alternative medicine J R Soc Med 1994 87 523 525 7932459 PMC1294769

[B43] Hirschkorn KA Bourgeault IL Conceptualizing mainstream health care providers’ behaviourism relation to complementary and alternative medicine Soc Sci Med 2005 61 157 170 10.1016/j.socscimed.2004.11.048 15847969

[B44] Clerc I Ventelou B Guerville M-A Paraponaris A Verger P General practitioners and clinical practice guidelines: a re-examination Medical care research and review 2011 68 504 518 10.1177/1077558710397761 21536601

[B45] Langworthy JM Birkelid J General practice and chiropractic in Norway: how well do they communicate and what do GP’s want to know? J Manipulative Physiol Ther 2001 24 576 581 10.1067/mmt.2001.118983 11753331

[B46] Foster NE Barriers and progress in the treatment of low back pain BMC Med 2011 9 108 http://www.biomedicalcentral.com/1741-7015/9/108 10.1186/1741/-7015-9-108 21943396 PMC3192671

[B47] Bishop PB Wing PC Knowledge transfer in family physicians managing patients with acute low back pain: a prospective randomized control trial Spine J 2006 6 282 288 10.1016/j.spinee.2005.10.008 16651222

[B48] Breen A Carrington M Collier R Vogel S Communication between general and manipulative practitioners: a survey Complement Ther Med 2000 8 8 14 10812754

